# A haplotype variation affecting the mitochondrial transportation of hMYH protein could be a risk factor for colorectal cancer in Chinese

**DOI:** 10.1186/1471-2407-8-269

**Published:** 2008-09-23

**Authors:** Huimei Chen, Lizhi Xu, Qiufeng Qi, Yanweng Yao, Ming Zhu, Yaping Wang

**Affiliations:** 1 Department of Medical Genetics, Medical School, Nanjing University, Nanjing, PR China; 2 Jiangsu Key Lab of Molecular Medicine, Nanjing, PR China

## Abstract

**Background:**

The human MutY homolog (*hMYH*), a DNA glycolsylase involved in the excision repair of oxidative DNA damage, is currently studied in colorectal cancer (CRC). We previously demonstrated a haplotype variant c.53C>T/c.74G>A of *hMYH *(T/A) increasing the risk for gastric cancer in Chinese. However, most investigations on correlation between *hMYH *and CRC are conducted in Western countries and the underlying mechanism has been poorly understood.

**Methods:**

To determine whether the haplotype T/A variant of *hMYH *was related to colorectal carcinogenesis, we performed a case-control study in 138 colorectal cancer (CRC) patients and 343 healthy controls in a Chinese population. Furthermore, the C/G for wild-type, C/A or T/G for single base variant and T/A for haplotype variant *hMYH *cDNAs with a flag epitope tag were cloned into pcDNA3.1+ vector and transfected into cos-7 cell line. Their subcellular localizations were determined by immunofluorescence assay.

**Results:**

It was found that the frequency of haplotype variant allele was statistically higher in CRC patients than that in controls (*P *= 0.02, odds ratio = 5.06, 95% confidence interval = 1.26 – 20.4). Similarly, significant difference of heterozygote frequency was indicated between the two groups (*P *= 0.019), while no homozygote was found. In addition, immunofluorescence analysis showed that hMYH protein with haplotype T/A variation presented in both nucleus and mitochondria, in contrast to the wild-type protein only converging in mitochondria. However, neither of the single missense mutations alone changed the protein subcelluar localization.

**Conclusion:**

Although preliminarily, these results suggest that: the haplotype variant allele of *hMYH *leads to a missense protein, which partly affects the protein mitochondrial transportation and results as nuclear localization. This observation might be responsible for the increased susceptibility to cancers, including CRC, in Chinese.

## Background

Base excision repair (BER) is a major mechanism for the repair of DNA base damage by reactive oxygen species (ROS)[1]. The most stable product of oxidative DNA damage, 8-oxo-guanine (8-oxoG), tends to mispaire with adenine, which would lead to a transversion of G:C to T:A [2,3]. The MutY DNA glycosylase initiates the repair pathway by recognizing and removing the adenine misincorporated with 8-oxoG [4]. A series of biochemical studies demonstrate that the *E. coli *strain lacking *MutY *is a mutator for G:C to T:A transversions [5,6]. Moreover, it is recently discovered that the germline mutations of the human MutY homolog (*hMYH*) increase the susceptibility to develop colorectal cancers (CRC) associated with adenomatous polyposis [7,8].

Our previous study detected 2 heterozygous base pair substitutions in Chinese, c.53C>T and c.74G>A, in *hMYH *gene [9]. Further cloning-sequencing showed that the mutations occurred at the same allele (haplotype T/A variation). The frequency of variant allele in suspected hereditary gastric cancer patients was significantly higher than that in the control group, which indicated that the T/A haplotype might form a partial genetic basis for the familial GC susceptibility in Chinese population. Interestingly, similar mutants have only been described in East Asian region. Shinmura et al. [10] has reported the 2 somatic mutations of MYH gene from lung cancer tissues from Japanese. However, no more analysis was made to clarify whether these two occurred on a same allele. Kim et al. [11] identified the germline haplotype T/A variation in patients with familial adenomatous polyposis (FAP) and showed tentative association with the development of FAP in Korean population.

On the other hand, germline mutations of *hMYH *have been extensively studied as risk factors for sporadic CRC in Caucasian populations [7,12,13]. In China, CRC has remained the fifth most common cancer and its morbidity has risen rapidly in recent years [14]. Based on the association regarding *hMYH *mutation and colorectal tumours, we therefore hypothesized that the haplotype T/A variation might be related with the pathogenesis of CRC in Chinese.

In addition, amino acid sequence analysis of hMYH protein illustrates that the haplotype T/A substitutions is predicted to generate missense mutations of p.Pro18Leu and p.Gly25Asp, respectively, and then mapped near to the functional N-terminal mitochondrial targeting sequences (MTS) domain [15,16]. This targeting sequence has been widely studied with a focus on mitochondrial transportation of protein, which is required for maintenance of the mitochondrial DNA repair capacity and genome stability [17,18]. Increasing evidences have suggested that mitochondrial oxidative damage contributed to human diseases, such as Alzheimer's disease, diabetes and cancer [19-22]. To elucidate the functional consequence of the haplotype T/A variation of *hMYH*, we constructed the recombinant cDNA with mutations, expressed them in cultured cos-7 cells and checked the mutant protein subcellular localization, and thereby investigated whether the haplotype variant of *hMYH *gene was associated with human colorectal carcinogenesis.

## Methods

### Patients and controls

A total of 138 Chinese patients with sporadic CRC had been enrolled from Jiangsu, China. CRC was diagnosed by histopathological examination using established clinical criteria and the clinical stage was evaluated on the basis of the TNM classification system of the UICC [23]. The distribution of CRC was categorized into three segments: the proximal colon (cecum, ascending colon, hepatic flexure, transverse colon), the distal colon (splenic flexure, descending colon, sigmoid colon), and the rectum above the anal canal. It was confirmed that these patients were not familial cases after the structured assessment including documentation of family history. Three hundred forty-three healthy individuals without any apparent cancer phenotype or history in the same geographic origin were taken as the control group. The demographic features and clinical manifestations of the subjects were shown in Table 1. No characteristic difference was observed in association between the cases and controls. Total genomic DNA was extracted from peripheral blood lymphocytes using QIAamp DNA blood mini kit (Qiagen, Hilden, Germany) according to the manufacturer's instructions. Informed consents were obtained from all the subjects and the study was approved by the ethics committee of the Nanjing University School of Medicine.

**Table 1:** Demographic features and clinical manifestations of CRC patients and normal controls.

	Normal controls	CRC patients
	(n = 343)	(n = 138)
Age, years	57.9 ± 14.5	59.6 ± 13.6
Gender (male/female [%])	207/136 (60.3/39.7)	85/53 (61.6/38.4)
Location	--	
Proximal colon		16 (11.6)
Distal colon		44 (31.9)
Rectum		78 (56.5)
TNM stages	--	
T-stage (%)		
T1		3 (2.17)
T2		33 (23.9)
T3		88 (63.8)
T4		14 (10.1)
N-stage (%)		
N0		71 (51.4)
N1		44 (31.9)
N2		23 (16.7)
M-stage (%)		
M0		115 (83.3)
M1		23 (16.7)

### Mutation screening in hMYH gene

The primers were designed to amplify the exon 2 of *hMYH *gene, where the T/A haplotype was located in: forward, 5'- AGCTATCACCCTTGGAAGGC -3', and reward, 5'- GTCTTGATACGTATCACAATCC -3'. The PCR products were tested by denaturing high-performance liquid chromatography (DHPLC) (WAVE system, USA) [24]. For the samples presenting an aberrant peak on DHPLC analysis, the nucleotide sequence was determined by direct sequencing of PCR products (ABI 3100 DNA sequencer, Applied Biosystems). The repeated PCR product would be cloned into the PMD-18-T vector (Takara) and amplified in *E. Coli *top10 for further sequencing analysis, if the two variations occurred in a sample.

### Construction of expression vectors

The wild-type *hMYH *(type 1) cDNAs, presented by Prof. Haruhiko Sugimura of the Hamamatsu University School of Medicine, was C-terminally tagged with a FLAG sequence and introduced into a mammalian expression vector pcDNA3.1+ [25]. Overlapping PCR site-directed mutagenesis was used to construct the haplotype T/A variation and either of the single base pair substitutions, T at nucleotide 53 or A at 74, into *hMYH *cDNA. This method was performed in two steps [26]: 1) complementary oligodeoxyribonucleotide primers were used to generate two DNA fragments (named "a" and "b") having overlapping ends; and 2) these fragments were combined in a subsequent 'fusion' reaction in which the overlapping ended anneal. Specific alterations in the nucleotide sequence were introduced by incorporating nucleotide changes into the overlapping oligo primers. The olignonucleotides designed for fragments "a" and "b" in the first PCR were listed in Table 2. The mutant constructions were confirmed by sequencing (ABI 3100, USA).

**Table 2:** Oligonucleotides used to create point mutations in the hMYH-type1-Flag/pcDNA

Mutation	Oligos for fragments "a" and "b" in the 1st. PCR (5'→3') *
p.Pro18Leu- p.Gly25Asp c.53C>T-c.74G>A	Fa:GGCGTGGATAGCGGTTTGA Ra:CACTTCCCACGGCTGCTCG**TaG**CTTCCTCATGA Fb: CGAGCAGCCGTGGGAAGT**GaT**CACAGGAAGCA Rb: TGAAATTCCTCCTGCGTCAGC
p.Pro18Leu c.53C>T	Fa:GGCGTGGATAGCGGTTTGA Ra:CACTTCCCACGGCTGCTCG**TaG**CTTCCTCATGA Fb:TCATGAGGAAG**CtA**CGAGCAGCCGTGGGAAGTG Rb: TGAAATTCCTCCTGCGTCAGC
p.Gly25Asp c.74G>A	Fa:GGCGTGGATAGCGGTTTGA Ra: TGCTTCCTGTG**AtC**ACTTCCCACGGCTGCTCG Fb: CGAGCAGCCGTGGGAAGT**GaT**CACAGGAAGCA Rb: TGAAATTCCTCCTGCGTCAGC

*Three bolded letters in the sequence represent the code of a mutated amino acid.

The lowercase letters are the substituted nucleotide residues.

### Immunofluorescence analysis

The expression vector was transfected into the cos-7 cell line, cultured on the slide glass, with the LipofectAMINE 2000 reagent (Invitrogen, USA). After 24 h, the cells were washed with PBS and fixed with methanol at -20°C for 30 min. The cells were subsequently washed once, treated with 0.25% Triton X-100 in PBS for 5 min, washed twice, and incubated with anti-FLAG M2 polyclone antibody (Sigma, USA) 40 μg/ml, at 4°C for overnight. Indirect immunofluorescence labelling was performed with a Fluorescence-5-isothiocyanate (FITC)-conjugated anti-mouse IgG second antibody (Sigma, USA) at room temperature for 60 min, and the mitochondria was stained with MitoTracker Red CMXros (Molecular Probes, USA). Fluorescence images were collected and analyzed by laser scanning confocal microscopy (LSM510, Zeiss).

### Statistical analysis

The SPSS 11.0 program was used to conduct the statistical analysis. For testing of significance of differences between 138 CRC patients and 343 healthy controls, nonparametrical Mann-Whitney Utest (unpaired) was applied. The observed genotype frequencies were compared with a chi-square 'goodness-of-fit' test to determine whether they were in Hardy-Weinberg equilibrium. The Fisher's exact tests were used to assess the genotype and allele distribution between two groups. The genotypic-specific risks were estimated as odds ratio (OR) with associated 95% confidence intervals (CI) by unconditional logistic regression and the ORs were adjusted for age and sex. For the statistical calculations, wild-type genotypes were assigned as "0" and heterozygous variant genotypes as "1". A P value < 0.05 was considered statistically significant.

## Results

### Association analysis of the c.53C>T/c.74G>A variation with sporadic CRC

**Figure 1:**
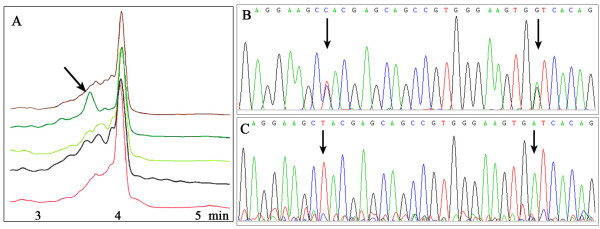
**Identification of haplotype variation c.53C>T-c.74G>A in *hMYH *gene.** (A) DHPLC chromatogram of exon 2 of *hMYH *gene showed the wild type and the mutant pattern. The arrow was pointed to the aberrant peak in patients with variant. (B) Partial sequence of exon 2 of *hMYH *gene (forward sequencing). Direct sequencing depicted 2 nucleotide substitutions (c.53C > T and c.74G > A). (C) Sequencing of cloned PCR product revealed the 2 nucleotide substitutions C > T and G > A on the same allele. Arrows showed the mutant sites.

An abnormal DHPLC peak was found in exon 2 of *hMYH *gene. The PCR product with aberrant elution profiles in DHPLC were sequenced directly and showed 2 heterozygous substitutions c.53C > T and c.74G > A (Figure 1A &1B). Repeated PCRs were cloned and transfected into E. Coli top10. Ten randomly picked clones for each sample were subjected to sequence and revealed that both of the substitutions affected a same allele with a wild-type allele remained (Figure 1C).

A total of 138 CRC patients and 343 healthy controls were screened for the haplotype variation (Table 1). The allele and genotype frequencies among controls were consistent with the Hardy-Weinberg equilibrium (*P *> 0.05). The heterozygous frequencies of the *hMYH *mutation were detected at 4.35% in CRC group and 0.87% in control (Table 3), while no homozygote was found in the present study.

The frequency of the variant allele detected in CRC patients (2.17%) was significantly higher than that in healthy individuals (0.44%), (*P *= 0.020, OR = 5.06 and 95% CI = 1.26 – 20.4). Table 3 also showed that the frequency of heterozygous *hMYH *genotype was statistically different between CRC patients and healthy controls (*P *= 0.019). Moreover, the heterozygous haplotype T/A variant genotype was not significantly associated with any clinical characteristics of CRC, while 5/6 CRC patients carrying this variant had a cancer at rectum.

**Table 3:** Distribution of the *hMYH *haplotype c.53C>T-c.74G>A mutation in the cases and controls.

	Control (%)	Case (%)	*P *value	OR (95% CI)
				
	No. (%)	No. (%)		
Genotype				
Wide-type genotype	340 (99.1%)	132 (95.7%)	0.019	5.15 (1.27–20.9)
Heterozygous genotype	3 (0.87%)	6 (4.35%)		

Allele				
Wild-type allele	683 (99.6%)	270 (97.8%)	0.020	5.06 (1.26–20.4)
Variant allele	3 (0.44%)	6 (2.17%)		

### Distinct subcellular localization of wild- and variant-type hMYH protein

**Figure 2:**
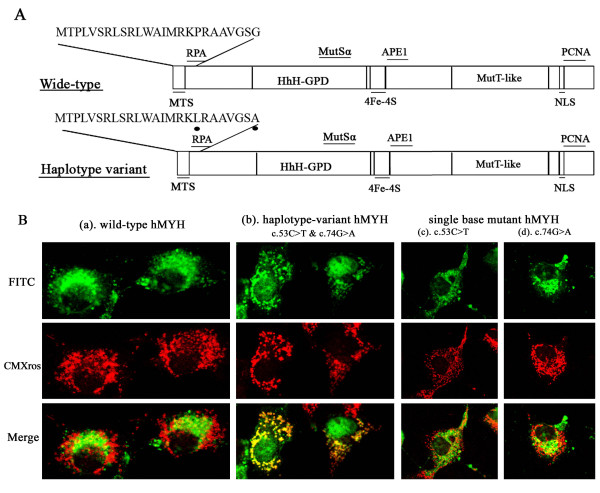
**Differences in structure and subcellular localization between the wild- and variant-type hMYH proteins. **(A) Schematic diagrams showed the structures of the wild-type hMYH type 1 protein (upper) and the hMYH protein with haplotype variation predicted a missense protein of p.Pro18Leu – p.Gly25Asp (lower). Dots represented the polymorphic amino acids, which was mapped to the N-terminus of the MTS domain. (B) The distinct subcellular localization of the wild- and variant-type of hMYH proteins. The wild-type (a) and variant-types (b, c, d) hMYH with the FLAG epitope tag at the C-terminus were expressed in cos-7 cells and stained with anti-FLAG M2 as the first antibody and FITC-conjugated anti-mouse IgG as the second antibody. The immunofluorescence microscopic image of FITC (green)-stained cells showed the localization of exogenous hMYH protein. The mitochondrias were counterstained with CMXros (red). The merged FITC and PI stained images showed overlapping yellow signals in the mitochondrias. Only the hMYH protein with haplotype variation (b) presented dual localization in nuclei and mitochondrias.

The transcript of *hMYH *gene detected in the cases with the haplotype variant encoded a missense protein of p.Pro18Leu/p.Gly25Asp, resulting in the substitutions c.53C>T/c.74G>A, located near to the functional N-terminal MTS sequence, as shown in Figure 2A. The hMYH type 1 protein was reported to be focused in the cell mitochondria [27]. We went on to investigate whether the structural changes actually affected its subcellular localization.

Overlapping PCR was used to construct three mutants from *hMYH *cDNA (type 1), the complex mutation c.53C>T/c.74G>A, and either of the single base pair substitution, c.53C>T or c.74G>A, in *hMYH*. Three kinds of mutants and wild-type *hMYH *cDNA were cloned into the pcDNA3.1 mammalian expression vector, with the FLAG sequence at the C-terminus, and transfected into cos-7 cell line respectively. We checked the fluorescence microscopic images of the transfected cells to identify the subcellular localization of the exogenous hMYH-FLAG proteins.

Consistent with the results previously reported [27], wild-type protein of hMYH was localized in the mitochondria (shown in Figure 2B–a). However the mutant protein with p.Pro18Leu/p.Gly25Asp has changed its subcellular localization and rather presented both in the nucleus and mitochondria of the cos-7 cells (Figure 2B–b). Interestingly, both of the two single base mutant hMYH-FLAG protein remained to be mitochondrial localized (Figure 2B–c &2B–d). It suggested that the haplotype variation of *hMYH *affected the localization of the gene product from the mitochondrial to the dual localization, and the protein transportation of the T/A variant-type hMYH protein was partly impaired, whereas single base mutant types were free from the influence.

## Discussion

ROS is the most prevalent source of DNA lesions in aerobic organisms, and mammalian cells have developed several repair pathways to cope with the resultant oxidative DNA damage [28]. The BER protein hMYH is essential in protecting against such damage and inherited defects within the protein lead to predisposition to carcinogenesis in humans [1,29]. Our previous study have demonstrated that a haplotype variation of *hMYH *(c.53C>T-c.74G>A, T/A) might be a genetic factor for the gastric cancer susceptibility in Chinese [9].

CRC is one of the major causes for cancer mortality in the world, and is becoming more prevalent in Asian countries [14]. In China, the incidence of CRC was initially low, but in recent years, the rate is increasing due to the changes of life style and nutritional habits. According to the report of the Health Ministry of China in 2002, the incidence rate of CRC is approx 0.037% and is the fifth leading cause of cancer mortality [30]. CRC is a collective term for cancers of the colon and rectum. Most of CRC occurs at colon in Caucasian, while the rectal cancer appears a higher frequency in Chinese. In the present study, the percentage of cancer was 11.6% in the proximal colon, 31.5% in the distal colon, and 56.5% in the rectum, respectively. Although the issue whether colon and rectal cancer should be considered as a single or two distinct entities is still being debated [31], cancers of the colon and rectum share many common features [32] and show little different in our results, and thus were discussed together in this study.

The heterozygous T/A haplotype variant configuration was discovered in 6/138 (4.35%) sporadic CRC cases and 3/343 (0.87%) controls. The frequency of variant T/A haplotype in patients was significantly higher than that in the control group (*P *= 0.019). It suggested a correlation between such *hMYH *variation and sporadic CRC susceptibility in Chinese. As oxidative DNA damage plays an important role in the carcinogenesis, we proposed that the variation of the BER gene *hMYH *might be commonly associated with the processes. It should be pointed out that we could not formally exclude the possibility that selection bias contributed to the observed differences between the two groups, due to a low frequency of this variant allele found.

Eukaryotic cells have nuclear and mitochondrial genomes and thus the cells have necessarily to develop either two distinct repair enzymes or a transport system to deliver the same enzymes into separate organelles [25,33]. The human *MutY *homolog comprises the entire *MutY *sequence flanked by extended N- and C-terminal domains, which are involved in subcellular targeting of hMYH: e.g. MTS and nuclear localization sequence (NLS) to the N- and C-terminus, respectively (Figure 2A) [34]. It suggests that the same hMYH protein presents differential subcellular localizations in human cells.

Several lines of evidence have shown that hMYH protein is a nuclear protein, preventing mutations from oxidative damage in the nuclei [4,35,36]. Takao et al. [17] detected that the C-terminus of hMYH was a functional NLS, which could target the heterologous hMYH protein into the nucleus. However, the full-length hMYH (type 1) with both NLS and MTS displayed rather a mitochondrial distribution. An alternative spliced form of hMYH (type 2) missing the first exon, pointed to MTS at N-terminus of hMYH, presented a nuclear localization [37]. It suggested that the MTS could be functionally dominant over the NLS in hMYH transportation. Tsai-Wu et al. [36] once proposed that hMYH was a shuttling enzyme with a steady-state nuclear distribution. Upon induction, presumably elevated oxidative potential in the mitochondria, MTS of hMYH presented its dominant function, and some unidentified mitochondrial factors would recognize this domain and transport hMYH from the nuclei to the mitochondria.

As it has been described above, the type 1 transcription of hMYH is concentrated in the mitochondria in cos-7 cells and recombinant hMYH has essentially similar activities to the partially purified protein [36]. This allows us to analyze the role of variation on MTS domain in the cos-7 cell model. We found that the hMYH protein with both missense mutations, that we detected previously, was dually localized in nucleus and mitochondria, and not focused in the mitochondria any more [38]. It indicated that the mutation affected the ability of hMYH protein to be transported into mitochondria. One rational explanation for it is that the p.Pro18Leu – p.Gly25Asp missense mutation in the N-terminus of hMYH influences the function of MTS domain, and then the MTS domain is difficult to be recognized. As a result, a proportion of the mutative hMYH protein would not be transported into mitochondria, and display the nuclear distribution instead [36]. Interestingly, immunofluorescence showed that the hMYH proteins with single missense mutation, p.Pro18Leu or p.Gly25Asp, remained in the mitochondria, similar to the wild-type protein. It seems that the effect of the mutations are additive and either one of the missense mutations in the haplotype is not enough to impair the function of MTS domain.

Mitochondrial oxidative energy metabolism is the major intracellular source of ROS and mitochondrial biomolecules including mitochondrial DNA are constantly exposed to a high level of ROS [39]. Almost all the studies performed to date have found increased oxidative damage in mitochondrial DNA and it is consistent with the observation that mitochondrial DNA mutations accumulate [40]. Mitochondrial DNA mutations have been reported in 10% to 70% of CRC and the presence of tumour mitochondrial DNA mutations seems to be a prognostic marker and a relevant predictive factor of CRC [21]. On the other hand, mitochondrial DNA repair enzymes involved in BER system play an important role in mitochondrial genome stability and hMYH protein is the requisite enzymes in this system [18,41,42]. Thus, it is possible that the haplotype variant allele of *hMYH *impairs the ability of oxidative damage repair in mitochondria and increases the instability of the mitochondrial genome, thus contributes to the carcinogenesis, including CRC. However, the glycolsylase activation of hMYH protein in mitochondria has not been directly analyzed in this study. Another point should be mentioned that, as no homozygous variation was detected in the population investigated, the impact of the heterozygous variant genotype on the *hMYH *gene will be explored in the following study.

## Conclusion

In conclusion, the *hMYH *variation affecting protein transportation is likely to associate with cancer susceptibility. A portion of hMYH protein arising from the haplotype allele was not able to be transported into mitochondria, and the variation might be responsible for the increased risk for developing CRC. We are aware that these data are still preliminary due to limited sample size. Furthermore, the adenine glycosylase activity of the mutants in mitochondria and mitochondrial genome stability under the influence of the mutants have not been directly investigated. Thus, validation of these results on larger cohorts and further functional studies are imperatively necessary.

## Abbreviations

hMYH: human MutY homolog; CRC: colorectal cancer; MTS: mitochondrial targeting sequences; BER: base excision repair; DHPLC: denaturing high-performance liquid chromatography.

## Competing interests

The authors declare that they have no competing interests.

## Authors' contributions

YP Wang conceived of the study and its design and was in charge of its coordination. HM Chen carried out the whole study, participated in data analysis and drafted the manuscript. LZ Xu performed cell culture and vector transfection. QF Qi carried out the genetic analysis and participated in data analysis. YW Yao carried out samples collection. M Zhu participated in vector construction.

## Pre-publication history

The pre-publication history for this paper can be accessed here:



## References

[B1] Hitomi K, Iwai S, Tainer JA (2007). The intricate structural chemistry of base excision repair machinery: implications for DNA damage recognition, removal, and repair. DNA Repair (Amst).

[B2] Kino K, Sugiyama H (2005). UVR-induced G-C to C-G transversions from oxidative DNA damage. Mutat Res.

[B3] Cunningham RP (1997). DNA glycosylases. Mutat Res.

[B4] McGoldrick JP, Yeh YC, Solomon M, Essigmann JM, Lu AL (1995). Characterization of a mammalian homolog of the Escherichia coli MutY mismatch repair protein. Mol Cell Biol.

[B5] Michaels ML, Cruz C, Grollman AP, Miller JH (1992). Evidence that MutY and MutM combine to prevent mutations by an oxidatively damaged form of guanine in DNA. Proc Natl Acad Sci USA.

[B6] Lu AL, Bai H, Shi G, Chang DY (2006). MutY and MutY homologs (MYH) in genome maintenance. Front Biosci.

[B7] Al-Tassan N, Chmiel NH, Maynard J, Fleming N, Livingston AL, Williams GT, Hodges AK, Davies DR, David SS, Sampson JR (2002). Inherited variants of MYH associated with somatic G:C-->T:A mutations in colorectal tumors. Nat Genet.

[B8] Sampson JR, Dolwani S, Jones S, Eccles D, Ellis A, Evans DG, Frayling I, Jordan S, Maher ER, Mak T (2003). Autosomal recessive colorectal adenomatous polyposis due to inherited mutations of MYH. Lancet.

[B9] Zhang Y, Liu X, Fan Y, Ding J, Xu A, Zhou X, Hu X, Zhu M, Zhang X, Li S (2006). Germline mutations and polymorphic variants in MMR, E-cadherin and MYH genes associated with familial gastric cancer in Jiangsu of China. Int J Cancer.

[B10] Shinmura K, Yamaguchi S, Saitoh T, Kohno T, Yokota J (2001). Somatic mutations and single nucleotide polymorphisms of base excision repair genes involved in the repair of 8-hydroxyguanine in damaged DNA. Cancer Lett.

[B11] Kim DW, Kim IJ, Kang HC, Jang SG, Kim K, Yoon HJ, Ahn SA, Han SY, Hong SH, Hwang JA (2007). Germline mutations of the MYH gene in Korean patients with multiple colorectal adenomas. Int J Colorectal Dis.

[B12] Halford SE, Rowan AJ, Lipton L, Sieber OM, Pack K, Thomas HJ, Hodgson SV, Bodmer WF, Tomlinson IP (2003). Germline mutations but not somatic changes at the MYH locus contribute to the pathogenesis of unselected colorectal cancers. Am J Pathol.

[B13] Kastrinos F, Syngal S (2007). Recently identified colon cancer predispositions: MYH and MSH6 mutations. Semin Oncol.

[B14] Li S, Nie Z, Li N, Li J, Zhang P, Yang Z, Mu S, Du Y, Hu J, Yuan S (2003). Colorectal cancer screening for the natural population of Beijing with sequential fecal occult blood test: a multicenter study. Chin Med J (Engl).

[B15] Ma H, Lee HM, Englander EW (2004). N-terminus of the rat adenine glycosylase MYH affects excision rates and processing of MYH-generated abasic sites. Nucleic Acids Res.

[B16] Ohtsubo T, Nishioka K, Imaiso Y, Iwai S, Shimokawa H, Oda H, Fujiwara T, Nakabeppu Y (2000). Identification of human MutY homolog (hMYH) as a repair enzyme for 2-hydroxyadenine in DNA and detection of multiple forms of hMYH located in nuclei and mitochondria. Nucleic Acids Res.

[B17] Takao M, Aburatani H, Kobayashi K, Yasui A (1998). Mitochondrial targeting of human DNA glycosylases for repair of oxidative DNA damage. Nucleic Acids Res.

[B18] Wang AL, Lukas TJ, Yuan M, Neufeld AH (2008). Increased mitochondrial DNA damage and down-regulation of DNA repair enzymes in aged rodent retinal pigment epithelium and choroid. Mol Vis.

[B19] Greaves LC, Taylor RW (2006). Mitochondrial DNA mutations in human disease. IUBMB Life.

[B20] Green K, Brand MD, Murphy MP (2004). Prevention of mitochondrial oxidative damage as a therapeutic strategy in diabetes. Diabetes.

[B21] Lievre A, Chapusot C, Bouvier AM, Zinzindohoue F, Piard F, Faivre J, Laurent-Puig P (2004). Mitochondrial DNA mutations in colorectal cancer a prognostic and a predictive factor of response to adjuvant chemotherapy. J Clin Oncol.

[B22] Reddy PH (2006). Mitochondrial oxidative damage in aging and Alzheimer's disease: Implications for mitochondrially targeted antioxidant therapeutics. J Biomed Biotechnol.

[B23] Sobin LHWC, editors (2002). UICC: TNM Classification of Malignant Tumors.

[B24] Gross E, Arnold N, Goette J, Schwarz-Boeger U, Kiechle M (1999). A comparison of BRCA1 mutation analysis by direct sequencing, SSCP and DHPLC. Hum Genet.

[B25] Yamaguchi S, Shinmura K, Saitoh T, Takenoshita S, Kuwano H, Yokota J (2002). A single nucleotide polymorphism at the splice donor site of the human MYH base excision repair genes results in reduced translation efficiency of its transcripts. Genes Cells.

[B26] Ho SN, Hunt HD, Horton RM, Pullen JK, Pease LR (1989). Site-directed mutagenesis by overlap extension using the polymerase chain reaction. Gene.

[B27] Ichinoe A, Behmanesh M, Tominaga Y, Ushijima Y, Hirano S, Sakai Y, Tsuchimoto D, Sakumi K, Wake N, Nakabeppu Y (2004). Identification and characterization of two forms of mouse MUTYH proteins encoded by alternatively spliced transcripts. Nucleic Acids Res.

[B28] Evans MD, Dizdaroglu M, Cooke MS (2004). Oxidative DNA damage and disease: induction, repair and significance. Mutat Res.

[B29] David SS, O'Shea VL, Kundu S (2007). Base-excision repair of oxidative DNA damage. Nature.

[B30] Li M, Gu J (2005). Changing patterns of colorectal cancer in China over a period of 20 years. World J Gastroenterol.

[B31] Li M, Li JY, Zhao AL, Gu J (2007). Colorectal cancer or colon and rectal cancer? Clinicopathological comparison between colonic and rectal carcinomas. Oncology.

[B32] Shin Y, Kim IJ, Kang HC, Park JH, Park HW, Jang SG, Lee MR, Jeong SY, Chang HJ, Ku JL (2004). A functional polymorphism (-347 G -> GA) in the E-cadherin gene is associated with colorectal cancer. Carcinogenesis.

[B33] Sutton MD, Walker GC (2001). Managing DNA polymerases: coordinating DNA replication, DNA repair, and DNA recombination. Proc Natl Acad Sci USA.

[B34] Nakabeppu Y (2001). Regulation of intracellular localization of human MTH1, OGG1, and MYH proteins for repair of oxidative DNA damage. Prog Nucleic Acid Res Mol Biol.

[B35] Yeh YC, Chang DY, Masin J, Lu AL (1991). Two nicking enzyme systems specific for mismatch-containing DNA in nuclear extracts from human cells. J Biol Chem.

[B36] Tsai-Wu JJ, Su HT, Wu YL, Hsu SM, Wu CH (2000). Nuclear localization of the human mutY homologue hMYH. J Cell Biochem.

[B37] Takao M, Zhang QM, Yonei S, Yasui A (1999). Differential subcellular localization of human MutY homolog (hMYH) and the functional activity of adenine:8-oxoguanine DNA glycosylase. Nucleic Acids Res.

[B38] Parker A, Gu Y, Lu AL (2000). Purification and characterization of a mammalian homolog of Escherichia coli MutY mismatch repair protein from calf liver mitochondria. Nucleic Acids Res.

[B39] Ott M, Gogvadze V, Orrenius S, Zhivotovsky B (2007). Mitochondria, oxidative stress and cell death. Apoptosis.

[B40] Barja G, Herrero A (2000). Oxidative damage to mitochondrial DNA is inversely related to maximum life span in the heart and brain of mammals. FASEB J.

[B41] Croteau DL, Stierum RH, Bohr VA (1999). Mitochondrial DNA repair pathways. Mutat Res.

[B42] Bohr VA (2002). Repair of oxidative DNA damage in nuclear and mitochondrial DNA, and some changes with aging in mammalian cells. Free Radic Biol Med.

